# Fluoride Concentration and pH Levels in Non-/Low-Alcoholic Beers

**DOI:** 10.1007/s12011-025-04780-1

**Published:** 2025-08-15

**Authors:** Guillermo Tamayo-Cabeza, Adam B. Kelly, Frank Lippert

**Affiliations:** 1 Department of Dental Public Health and Dental Informatics, School of Dentistry, Indiana University, Indianapolis, IN USA; 2 School of Dentistry, Oral Health Research Institute, Indiana University, Indianapolis, IN USA

**Keywords:** Fluoride, PH, Acidity, Beer, Beverage

## Abstract

Non- or low-alcoholic beers (NLABs) have gained consumer popularity, yet limited data exist on their fluoride concentration and acidity. A convenience sample of 71 commercially available NLABs was sampled from two retail stores in Indianapolis, IN, USA. Fluoride concentration was measured using a fluoride ion-selective electrode, and pH levels were recorded with a calibrated pH meter. Spearman’s rank correlation assessed relationships between fluoride concentration and pH. Beverage styles were categorized into ten types: Amber Ale (*n* = 2), Golden Ale (*n* = 2), Golden Lager (*n* = 18), India Pale Ale (*n* = 23), Pale Ale (*n* = 4), sour (*n* = 5), sparkling hop water (*n* = 5), stout (*n* = 2), wheat (*n* = 5), and others. Fluoride concentrations ranged from 0.09 to 1.01 ppm (mean ± SD: 0.43 ± 0.24 ppm), with Amber Ales showing the highest mean value and sparkling hop water the lowest. All products had acidic pH values (range: 3.29 to 4.64). No significant correlations were found between fluoride concentration and pH. NLABs showed a wide range of fluoride concentrations and acidic pH values across styles. Future studies should aim to quantify the contribution of NLABs to total fluoride dietary intake and assess their role in erosive tooth wear using risk assessment methodologies.

**Supplementary Information:**

The online version contains supplementary material available at 10.1007/s12011-025-04780-1.

## Introduction

Fluoride is the ionic form of fluorine, a halogen element that occurs naturally in the environment and is commonly found in water, soil, and various foods in the human diet [1, 2]. Fluoride enhances the remineralization of dental enamel and contributes to dental caries prevention, supporting its widespread implementation in community-based fluoridation programs, including water, salt, and milk fluoridation [3]. Fluoride has also been incorporated into oral hygiene products (toothpastes and mouthwashes) for at-home use and professional products to be applied or prescribed by dental practitioners, such as varnishes, foams, gels, or prescription-strength toothpastes [4]. Current evidence supports fluoride’s mechanisms for dental caries prevention as mainly topical, and on a minor effect, systemic through low levels in the saliva [5]. However, despite fluoride’s proven protective effects against dental caries, higher intakes of fluoride during enamel development can cause dental fluorosis [6], which is characterized by hypomineralization of tooth enamel that can lead to detrimental effects on the form and function of the teeth. Furthermore, higher than optimal fluoride concentrations in water, especially in endemic areas with consumption of groundwater [7], are associated with skeletal fluorosis, a condition that weakens the bone tissue [8]. In recent decades, research has expanded beyond dental and skeletal effects of fluoride to other negative effects on human health, including neurotoxicity. For instance, higher prenatal fluoride exposure has been associated with lower cognition and adverse behavioral effects in the offspring [9–12]. These potential effects on health have increased the interest in understanding the contribution of various sources of fluoride in the total intake [13–15], especially for vulnerable populations.

Among dietary sources, some beverages can represent a potential source of fluoride intake [14, 16]. The present study evaluated fluoride concentrations in non- or low-alcoholic beers (NLABs), which are fermented or non-fermented drinks that contain little to no ethanol (typically ≤ 0.5% alcohol by volume), designed to mimic the attributes of traditional alcoholic beers while reducing or eliminating intoxicating effects [17]. A recent study reported that 28.44% out of 1906 surveyed adults in the USA endorsed having consumed a non-alcoholic beverage in the past year, with regular monthly use reported by those who also consume alcoholic beverages, which supports their increased popularity in recent years [18]. Among the reasons that may motivate the consumption of NLABs are health reasons (including pregnancy status), drinking and driving, and avoiding alcohol-related negative effects [19]. Compared to alcoholic beverages, there is limited knowledge on the fluoride concentration in NLABs in the literature. A higher fluoride concentration has been previously reported in alcoholic beverages with lower ethanol levels compared to those with higher content of alcohol [20]. In addition, fluoride concentrations in beer have been found to vary depending on the country of origin [16, 21, 22] and the water used in beer production, with levels exceeding 1.5 mg/L in the USA [22].

The acidity of beverages has been suggested as a contributing factor to erosive tooth wear [23]. Erosive tooth wear is a multifactorial condition driven by chemical and mechanical factors, leading to a dissolution of the minerals within the dental structure. Globally, erosive tooth wear has been considered a growing health problem, with prevalences in adults reaching up to 83% [24], with acid exposure either from intrinsic or extrinsic sources and dietary habits representing the main associated factors [25]. Previous studies have reported acidic or low pH values in beers with dental erosive potential [26], requiring further investigation of acidic levels in NLABs.

The objective of this study was to determine the fluoride concentration and pH levels of various commercially available NLABs in the USA. Specifically, the study aimed to (1) quantify fluoride concentrations and pH values in NLABs and (2) compare their fluoride concentrations and pH levels according to different beer styles.

## Methods

### Sample Collection

A convenience sample of all NLABs commercially available in two retail stores (total wine, Kroger) in Indianapolis, Indiana, USA, in January 2024 was chosen. Table 1 informs about the study sample and provides pertinent information, including brand, style, production category (craft or industrial; domestic or imported) and packing (either glass bottle or aluminum cans). All collected NLABs were stored under ambient conditions after purchase.

**Table 1:** Description of non-/low-alcoholic beverages included in the study by brand, production category, style, packaging, and sample count (*N* = 71)

Brand/brew	Production	Style	Packing
Athletic Brewing Co. Athletic Lite	Craft domestic	Golden Lager	can
Athletic Brewing Co. Cerveza Athletica	Craft domestic	Golden Lager	can
Athletic Brewing Co. Free Wave Hazy IPA	Craft domestic	India Pale Ale	can
Athletic Brewing Co. Run Wild IPA	Craft domestic	India Pale Ale	can
Athletic Brewing Co. Upside Dawn Golden	Craft domestic	Golden Ale	can
Beck’s Non-Alcoholic	Industrial imported	Golden Lager	bottle
Bitburger Premium Pils Alkoholfrei	Industrial imported	Golden Lager	bottle
Blue Moon Belgian White	Industrial domestic	Wheat	can
Bravus Brewing Co. Blood Orange IPA	Craft domestic	India Pale Ale	can
Bravus Brewing Co. West Coast IPA	Craft domestic	India Pale Ale	can
Brewdog Elvis Hoppy Grapefruit	Craft imported	India Pale Ale	can
Brewdog Hazy New England Style Hazy	Craft imported	India Pale Ale	can
Brewdog Nanny State Hoppy Golden	Craft imported	Golden Ale	can
Brewdog Punk Hoppy Pale	Craft imported	Pale Ale	can
Budweiser Zero	Industrial domestic	Golden Lager	bottle
Busch	Industrial domestic	Golden Lager	can
Ceria Brewing Co. Grainwave Belgian-Style White	Craft domestic	Wheat	can
Ceria Brewing Co. Indiewave IPA	Craft domestic	India Pale Ale	can
Clausthaler IPA Dry Hopped Non-Alcoholic	Industrial imported	India Pale Ale	bottle
Clausthaler ISO	Industrial imported	Other	can
Clausthaler Original Non-Alcoholic	Industrial imported	Golden Lager	bottle
Coors (Original) Coors Edge	Industrial domestic	Golden Lager	bottle
Corona El Sabor Mas Fino	Industrial imported	Golden Lager	bottle
Estrella Galicia Lager 0.0 Beer	Industrial imported	Golden Lager	can
Go Brewing Freedom Cali Ale	Craft domestic	Pale Ale	can
Go Brewing New School Sour	Craft domestic	Sour	can
Gruvi Golden	Craft domestic	Golden Lager	can
Gruvi Juicy IPA	Craft domestic	India Pale Ale	can
Gruvi Mocha Nitro Stout	Craft domestic	Stout	can
Guinness 0	Industrial imported	Stout	can
Hairless Dog Brewing Company Citra Lager	Craft domestic	Golden Lager	can
Hairless Dog Brewing Company IPA	Craft domestic	India Pale Ale	can
Lagunitas Brewing Co. Berry + Lemon	Industrial domestic	Sparkling Hop Water	can
Lagunitas Brewing Co. Blood Orange	Industrial domestic	Sparkling Hop Water	can
Lagunitas Brewing Co. Hoppy Refresher	Industrial domestic	Sparkling Hop Water	can
Lagunitas Brewing Co. IPA	Industrial domestic	India Pale Ale	bottle
O’Doul’s Amber	Industrial domestic	Amber Ale	bottle
O’Doul’s Golden	Industrial domestic	Golden Lager	bottle
Pabst Blue Ribbon Non-Alcoholic	Industrial domestic	Golden Lager	can
Partake Brewing Blonde	Craft imported	Golden Lager	can
Partake Brewing Dunkel	Craft imported	Other	can
Partake Brewing Hazy IPA	Craft imported	India Pale Ale	can
Partake Brewing IPA	Craft imported	India Pale Ale	can
Partake Brewing Pale	Craft imported	Pale Ale	can
Partake Brewing Peach Gose	Craft imported	Sour	can
Penn’s Best	Industrial domestic	Golden Lager	can
Samuel Adams Gold Rush	Industrial domestic	Golden Lager	can
Samuel Adams Just The Haze	Industrial domestic	India Pale Ale	can
Shorts Thirst Mutilator	Craft domestic	Sparkling Hop Water	can
Sierra Nevada Brewing Co. Hop Splash	Craft domestic	Sparkling Hop Water	can
Sierra Nevada Brewing Co. Trail Pass	Craft domestic	India Pale Ale	can
Sober Carpenter Irish Style Red	Craft imported	Other	can
Sober Carpenter White	Craft imported	Wheat	can
Stella Artois Liberte	Industrial imported	Golden Lager	bottle
Strive Juicy IPA	Craft imported	India Pale Ale	can
Sun King Brewery Hopopolis Non-Alcoholic IPA	Craft domestic	India Pale Ale	can
Surreal Brewing Co. Juicy Mavs Hazy IPA	Craft domestic	India Pale Ale	can
Surreal Brewing Co. Non-Alcoholic Kolsch Style	Craft domestic	Other	can
Two Roots Brewing Co. Helles Enough Said	Craft domestic	Other	can
Two Roots Brewing Co. IPA New West	Craft domestic	India Pale Ale	can
Untitled Art Citra Haze	Craft domestic	India Pale Ale	can
Untitled Art Juicy IPA	Craft domestic	India Pale Ale	can
Untitled Art Lychee Sorbet	Craft domestic	Sour	can
Urban Artifact Seedless Mango	Craft domestic	Sour	can
Urban Artifact Seedless Strawberry	Craft domestic	Sour	can
Weihenstephaner (Hefe Weissbier) Non-Alcoholic	Industrial imported	Wheat	bottle
WellBeing Heavenly Body Golden Wheat	Craft domestic	Wheat	can
WellBeing Hellraiser Dark Amber	Craft domestic	Amber Ale	can
WellBeing Intentional IPA	Craft domestic	India Pale Ale	can
WellBeing Light Match Day	Craft domestic	Pale Ale	can

### Fluoride Analysis

One mL of each sample was dispensed into an appropriately labeled vial. Then, 1 mL of total ionic strength adjustment buffer (TISAB) II was added to each sample vial and vortexed to ensure thorough mixing [16, 27]. Each sample was analyzed in duplicate for fluoride using a fluoride ion-selective electrode coupled to a pH/ISE meter (Orion™ Fluoride Electrode and Dual Star™ pH-meter, Thermo Scientific, Waltham, MA, USA). A calibration curve was generated using fluoride standards (Orion™ ISE calibration standards; 0.02, 0.1, 0.2, 1.0, and 5.0 ppm fluoride) following the same protocol. Millivolt measurements from the samples were collected, and the fluoride concentrations in NLAB samples were calculated based on the equation describing the relationship between the logarithm of the fluoride concentration of the standards and their corresponding millivolt values (*r*2 > 0.9).

### pH Determination

pH was measured on each sample using a pH meter (Accumet AR25). After calibrating using standard buffer solutions, the pH was measured for each NLAB sample.

### Statistical Analysis

Descriptive statistics were used to summarize fluoride concentration and pH levels across different beverage styles, with results presented as mean ± standard deviation (SD) and median (interquartile range (IQR)). Correlation analysis was performed using Spearman’s rank correlation coefficient to assess the relationships between fluoride concentration and pH, as these variables did not meet the assumptions required for Pearson’s correlation. A significance level of 0.05 was used for all statistical tests. Data analysis and visualization were conducted using R version 4.4.2.

## Results

A total of 71 products from 35 distinct commercial brands were identified (Table 1) and categorized into the corresponding beer/beverage types: Amber Ale (*n* = 2), Golden Ale (*n* = 2), Golden Lager (*n* = 18), India Pale Ale (*n* = 23), Pale Ale (*n* = 4), sour (*n* = 5), sparkling hop water (*n* = 5), stout (*n* = 2), wheat (*n* = 5), and other including isotonic, Irish style, and Dunkel (*n* = 5).

The overall fluoride concentration ranged from 0.09 to 1.01 ppm with mean ± SD of 0.43 ± 0.24 ppm and median (IQR) of 0.48 (0.41) ppm. The style of NLAB with the highest mean fluoride concentration (Table 2) was Amber Ale (mean ± SD: 0.73 ± 0.21 ppm), followed by Stouts (mean ± SD: 0.69 ± 0.20 ppm). Sparkling hop water showed the lowest mean fluoride concentration (mean ± SD: 0.16 ± 0.08 ppm) among the products analyzed (Table 2).

**Table 2:** Fluoride concentration (ppm) across different non-/low-alcoholic beverage styles (*N* = 71)

Style	Number	Mean ± SD	Median (IQR)	Min	Max
Amber Ale	2	0.73 ± 0.21	0.73 (0.15)	0.58	0.88
Golden Ale	2	0.37 ± 0.39	0.37 (0.28)	0.09	0.64
Golden Lager	18	0.53 ± 0.25	0.59 (0.34)	0.14	1.01
India Pale Ale	23	0.38 ± 0.20	0.39 (0.39)	0.10	0.80
Pale Ale	4	0.47 ± 0.25	0.58 (0.16)	0.10	0.62
Sour	5	0.57 ± 0.24	0.49 (0.36)	0.28	0.86
Sparkling hop water	5	0.16 ± 0.08	0.12 (0.03)	0.11	0.29
Stout	2	0.69 ± 0.20	0.69 (0.14)	0.55	0.83
Wheat	5	0.30 ± 0.18	0.37 (0.32)	0.10	0.48
Other*	5	0.34 ± 0.21	0.42 (0.36)	0.10	0.56

*Including isotonic (ISO), Irish style, and Dunkel

**Fig. 1:**
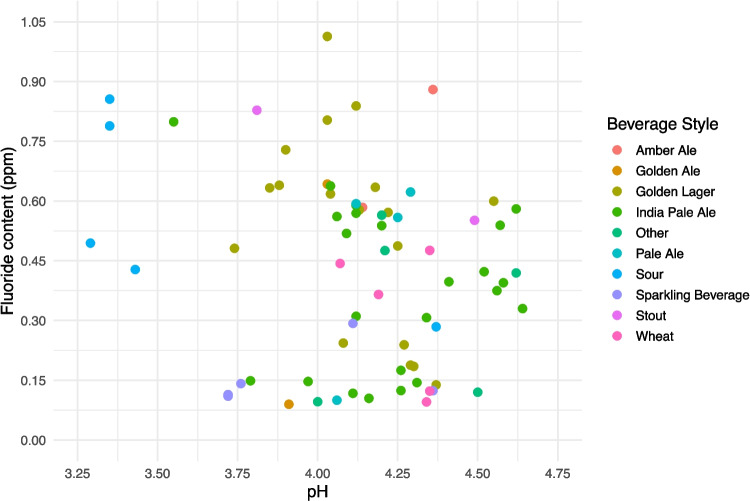
Relationship between fluoride concentration (ppm) and pH in non-/low-alcoholic beverages (*N* = 71; Spearman’s rank correlation rho: − 0.20; *p*-value: 0.09). Graphic created using the package ggplot2 [28]

The overall pH levels ranged from 3.29 to 4.64, indicating that all products analyzed were in the acidic range (Table 3). The mean ± SD and median (IQR) pH level across all products were 4.13 ± 0.31 and 4.14 (0.31), respectively. The style of non-/low-alcoholic beverage with the lowest mean pH was sour (mean ± SD: 3.56 ± 0.46) followed by sparkling hop water (mean ± SD: 3.93 ± 0.29). Figure 1 presents the relationship between fluoride concentration and pH level of the samples analyzed, with each point presenting an individual product, color-coded by beverage style. There was no significant correlation between fluoride concentration and pH level (Spearman’s rank correlation rho: − 0.20, *p-*value: 0.09).

**Table 3:** pH levels across different non-/low-alcoholic beverage styles (*N* = 71)

Style	Number	Mean ± SD	Median (IQR)	Min	Max
Amber Ale	2	4.25 ± 0.16	4.25 (0.11)	4.14	4.36
Golden Ale	2	3.97 ± 0.08	3.97 (0.06)	3.91	4.03
Golden Lager	18	4.12 ± 0.20	4.12 (0.23)	3.74	4.55
India Pale Ale	23	4.23 ± 0.28	4.20 (0.37)	3.55	4.64
Pale Ale	4	4.18 ± 0.11	4.19 (0.16)	4.06	4.29
Sour	5	3.56 ± 0.46	3.35 (0.08)	3.29	4.37
Sparkling hop water	5	3.93 ± 0.29	3.76 (0.39)	3.72	4.36
Stout	2	4.15 ± 0.48	4.15 (0.34)	3.81	4.49
Wheat	5	4.26 ± 0.13	4.34 (0.16)	4.07	4.35
Other*	5	4.31 ± 0.25	4.21 (0.30)	4.00	4.62

*Including isotonic (ISO), Irish style, and Dunkel

## Discussion

The present study aimed to determine the fluoride concentration and pH levels of a variety of NLABs. Fluoride concentrations of the tested NLABs did not exceed that of tap water in areas with community water fluoridation and were comparable to that of bottled water commercially available in the same location [29]. The US Department of Agriculture’s National Fluoride Database reported fluoride content in commonly consumed foods and beverages using nationally representative sampling and validated analytical methods [30]. Among alcoholic beverages, light and regular beers were found to contain a mean value of 0.45 ppm fluoride. In the present study, while the mean fluoride concentration of NLABs was 0.43 ppm, some samples exhibited values as high as 1.01 ppm. This finding is similar to other studies that have demonstrated differences in fluoride concentrations among beverages with lower and higher alcohol levels. For instance, in a comparison of fluoride concentration among drinks with varied levels of alcohol collected in Poland, it was found that drinks with the lowest level of alcohol contained the highest fluoride levels [20]. This observation may be explained by the greater contribution of water to the overall composition of low-alcohol beverages, wherein fluoride levels are influenced by the fluoride concentration of the water used during production [16].

Fluoride concentrations in beers have been demonstrated to differ significantly by country of origin [16], and those with USA origin have been reported to show higher concentrations [22]. Specifically, a study of fluoride concentrations in beers from several different countries of origin (including countries from Europea, North and South America, and east Asia) reported that samples with the highest concentrations were those of USA origin, with concentrations of 1.77 ppm [22]. The same study included non-alcoholic beers from Spain, Germany, Ireland, and the Netherlands, showing fluoride concentrations ranging from 0.13 to 0.76 ppm. In comparison, a maximum value of 1.01 ppm was observed in the present study for NLABs collected in the USA. In addition, when looking at specific beverage styles, sparkling hop waters showed the lowest mean fluoride concentration (0.16 ppm). In comparison, in a study of soft drinks collected in Spain, sparkling soft drinks had fluoride concentrations as high as 1.37 ppm [14]. These comparisons highlight the wide variability in fluoride content across countries and beverage types, emphasizing the importance of monitoring locally available products as potential sources of fluoride intake.

The NLABs included in this study showed pH levels (3.29 to 4.64) in the acidic range. This finding is similar to previous studies that have assessed the pH levels of various beverages in the USA. For instance, Reddy, Norris [31] found that among 380 beverages collected in the USA, which included beer-like beverages, 93% of samples analyzed for pH showed values below 4.0, and only 7% had pH ≥ 4.0. Their study highlighted the potential implications of the consumption of these beverages with lower pH for erosive tooth wear. The pH of extrinsic solutions from the diet (such as beverages with low pH) is considered one of the major factors for erosive tooth wear, a condition that has shown a prevalence of 83% in adults and can lead to significant loss of tooth structure, resulting in functional, aesthetic, and restorative complications [24]. Given their acidic nature, NLABs may represent a meaningful extrinsic source of dietary acid exposure and thus contribute to the multifactorial etiology of erosive tooth wear in adults. Future studies should investigate whether the consumption of NLABs is associated with erosive tooth wear outcomes, particularly through epidemiological designs that can account for beverage type and consumption patterns.

In the present study, no significant correlation was observed between fluoride concentration in NLABs and pH levels. This finding was similar to the results found by Buljac, Bralić [32], where pH had no influence on the fluoride concentration in the studied beers of Croatian producers. This lack of correlation is likely due to the fact that fluoride content remains stable throughout fermentation, whereas pH and alcohol levels fluctuate as byproducts of metabolic processes. Thus, the fluoride concentration in NLABs likely reflects the composition of the water and raw materials used, rather than changes occurring during fermentation.

Several limitations of this study should be acknowledged when interpreting the findings. First, the sample selection was a convenience sample drawn from two stores. This localized sample may not capture the full variability of fluoride and pH in all NLABs on the market. Products available in other regions or countries, or at different times, could have different fluoride levels, especially given that regional water fluoridation practices vary and can influence beverages made in those areas. Despite these limitations, this study included a diverse assortment of beverage types (ranging from ales and stouts to sours and sparkling drinks), which improves the robustness of the comparison across styles. Future studies could draw comparisons to alcoholic beers, other non-/low-alcoholic beverages (such as hard seltzers), and study consumption patterns among populations at risk of elevated fluoride exposure.

## Conclusions

Non- or low-alcoholic beers showed a wide range of fluoride concentrations and acidic pH values across styles. No significant correlations were found between fluoride and pH. Future studies should aim to quantify the contribution of NLABs to total fluoride dietary intake and assess their role in erosive tooth wear using risk assessment methodologies.

## Supplementary Information

Below is the link to the electronic supplementary material.

Supplementary Material 1 (XLSX 13.6 KB)

## Data Availability

The dataset generated during and analyzed during the current study is available in the Supplementary Material.
